# Giant Peripheral Ossifying Fibroma of the Posterior Mandible-A Rare Case Report

**Published:** 2017-10-01

**Authors:** Vandana Reddy, Arunkumar KV, Vijay Wadhwan, Arvind Venkatesh

**Affiliations:** 1 *Dept. of Oral Pathology and Microbiology, Subharti Dental College & Hospital, Meerut, India*; 2 *Dept. of Oral & Maxillofacial Surgery, Subharti Dental College & Hospital, Meerut, India*; 3 *Dept. of Oral Pathology & Microbiology, Subharti Dental College & Hospital, Meerut, India*; 4 *Dept. of Oral Pathology & Microbiology, Subharti Dental College & Hospital, Meerut, India*

**Keywords:** Giant, gingival neoplasms, gingival growths, ossifying fibroma

## Abstract

Large, atypical peripheral ossifying fibromas are known as giant peripheral ossifying fibromas. These lesions have often been associated with heterogeneous clinical and radiographic characteristics subsequently leading to their misdiagnosis. Biopsies have been the gold standard for the diagnosis of such lesions. This study reports on an acute presentation of giant peripheral ossifying fibroma, clinically mimicking a malignant lesion due to its atypical presentation along with its characteristic histological features, which led to the establishment of the diagnosis.

## Introduction

Peripheral Ossifying Fibromas (POFs) are focal overgrowths, occurring in the gingiva (1).They are also known as ossifying fibroid epulis, peripheral fibromas with calcifications, and calcifying fibroblastic granulomas(2). Clinically, these lesions appearas small, well-demarcated focal masses on the gingivawith a sessile or pedunculated base, usually originating from an interdental papilla (1, 2). The conventional lesionsare typically less than 2cm in size, however it has been recognized that some POFs may grow quite large and may displace the teeth. In such cases, non-typical clinical and radiographic appearance, presence of soft tissue calcifications may lead to misdiagnosis of the lesions (3). Such lesions are generally termed Giant Peripheral Ossifying Fibromas (GPOFs). 

This report presents a case of rapidly growing GPOF, clinically mimicking a malignant lesion due to its atypical presentation along with its characteristic histological features, which led to the establishment of the diagnosis.

## Case report

A 55-year-old North Indian male reported to theout-patient service of the current study with a complaint of a swelling in the right side of hisface for 20 days. History revealed that the swelling was initially the size of a pea, and grew rapidly to attain the present size. He also complained of mild pain and discomfort during mastication associated with the swelling. The patient’s medical history revealed that he was not currently under the care of a physician, had no known medical problems, and was not currently taking any medications. He had undergone dental extraction in the area associated with the swelling 6 months prior to referral. He reported that he wasa cigarette smoker, tobacco chewer, and alcoholic for 30 years. 

**Figure 1:**
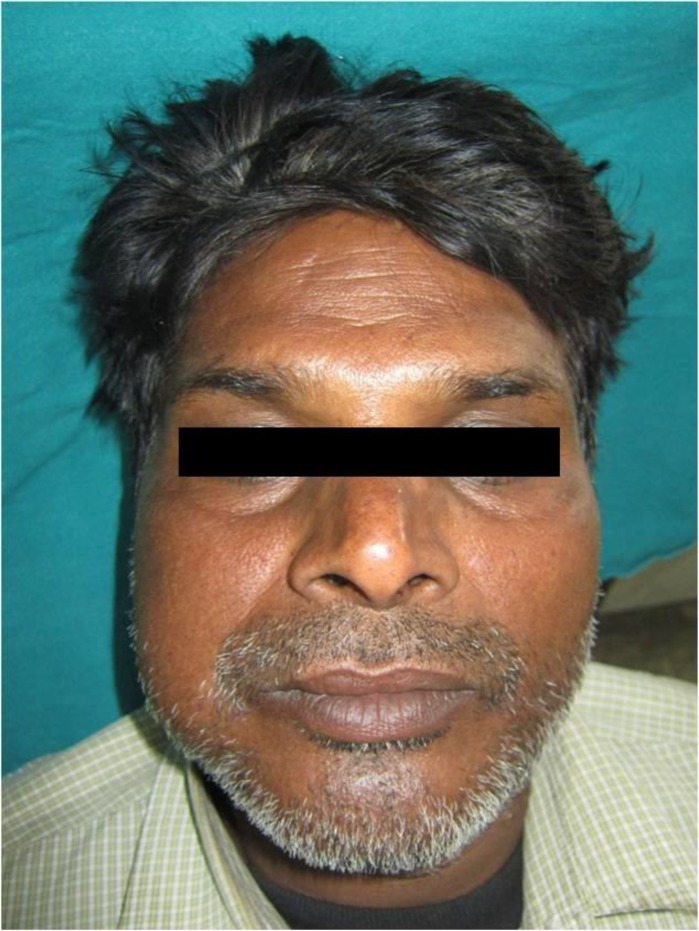
Clinical Picture Showing Diffuse Swelling of the Right Side of the Face

**Figure 2:**
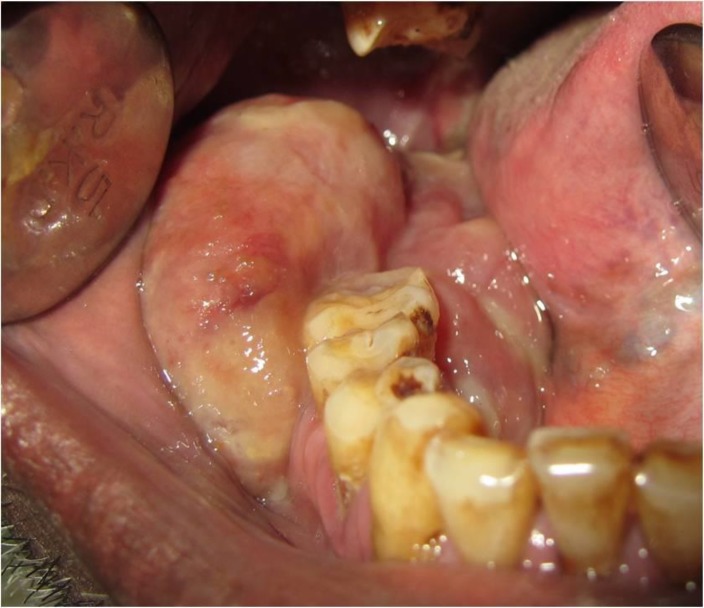
Intra Oral View: Pinkish, sessile, bilobed, fibrous soft tissue mass extending from region 45 to 48, involving both the buccal and lingual vestibule

**Figure 3:**
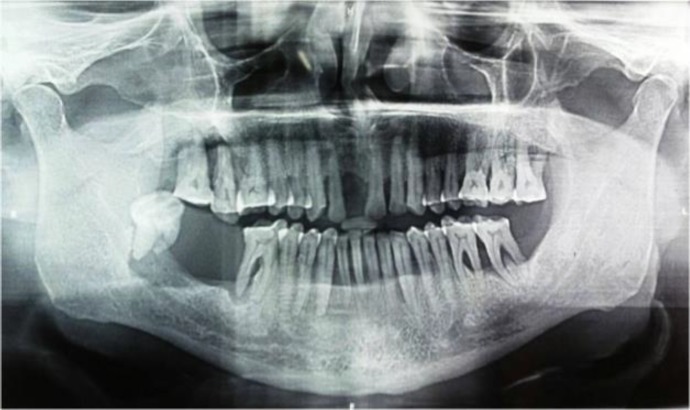
OPG Showing Soft Tissue Opacificationin the Right Posterior Mandible Extending From Region 46 to 48

**Figure 4:**
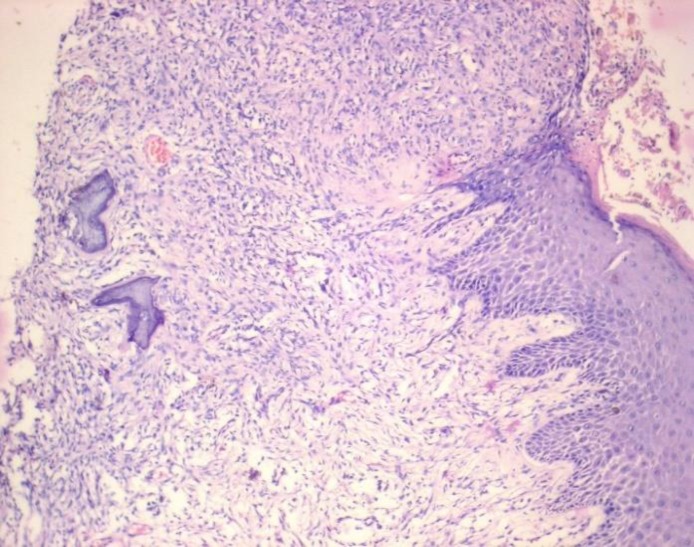
HyperplasticParakeratinized Stratified Squamous Epithelium with Areas of Ulceration, Covered With a Fibro-Purulent Membrane and a Subjacent Area of Granulation Tissue (H&E, original magnification x100

**Figure 5:**
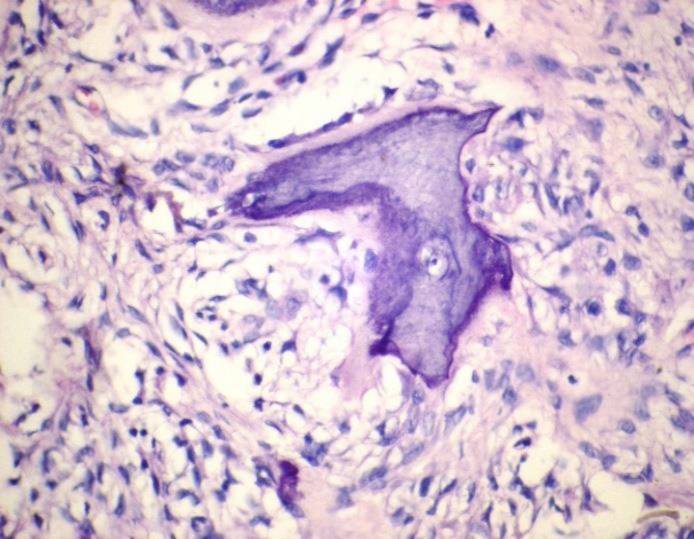
Areas Showing Cellular Fibroblastic Proliferation and Mineralization (H&E, original magnification x400

**Figure 6:**
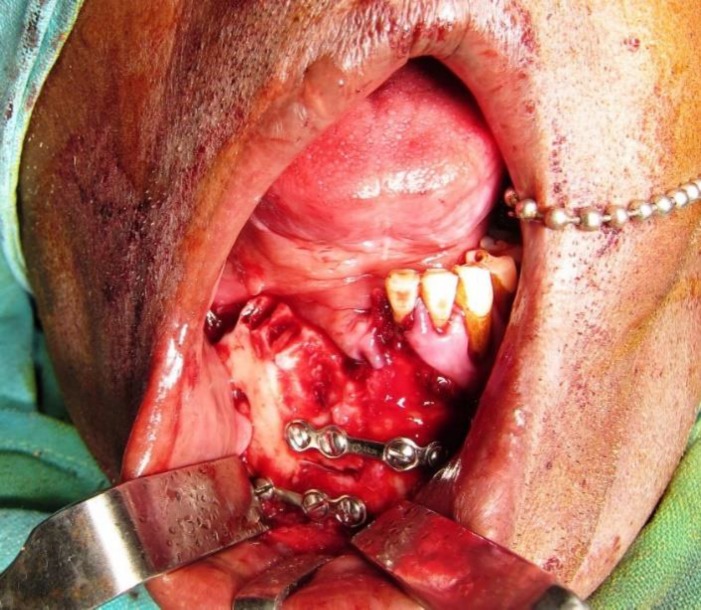
Post Excisional View Showing Access Step Osteotomy Site

Extra oral examination revealed a diffuse swelling of the right side of the face, measuring approximately 5x4 cm in posterior body and angle region of the mandible (Figure 1). There were no palpable submandibular and sublingual lymph nodes. Intra oral examination revealed a pinkish, sessile, bilobed, fibrous soft tissue mass extending distal to the right mandibular second premolar region involving both the buccal and lingual vestibule (Figure 2). The lobes were attached through the edentulous space in the alveolar ridge in relation to 47, which was extracted 6 months prior to referral, and there was considerable posterior displacement of 48, giving the lesion a dumb-bell shaped appearance. The buccal and lingual lobes measured approximately 50x40x30 mm and 50x20x20 mm, respectively. The growth was non-tender, non-compressible, non-fluctuant, and soft to firm in consistency. The mucosa over the growth appeared stretched. Oral pantomograph(Figure 3) revealed soft tissue opacification in the right posterior mandible, extending from region 46 to 48 with displacement of 48 and pressure resorption distal to 48. There was no root resorption in relation to 46 and 48. The radiograph also revealed generalized horizontal bone loss with multiple furcation involvement. Although, the clinical and radiographic appearance of the lesion at the timeof reporting hada benign appearance due to the absence of secondary ulcerations, invading margins, extensive bone loss or involvement of underlying structures, acute history and the extent of the lesion resulted in a wide range of differential diagnoses to allow a range from benign to aggressive malignant lesions. The differential diagnoses included peripheral myxoma, pleomorphic adenoma, mucoepidermoid carcinoma, aggressive fibromatosis, giant POF, nodular fasciitis, sarcomas, and metastatic carcinomas. An incisional biopsy was made from the growth in the buccal vestibule, which revealed hyperplastic parakeratinized stratified squamous epithelium, which was ulcerated at large, covered with a fibro-purulent membrane and a subjacent area of granulation tissue (Figure 4). Deeper areas showed highly cellular fibroblastic proliferation and associated mineralization, which consisted of mainly woven and trabecular type of bone along with areas showing dystrophic calcifications (Figure 5). Based on the histopathological features, a diagnosis of peripheral ossifying fibroma was made. An access step osteotomy was performed at the right body and alveolar region for better visualization and complete excision of the lesion, which had extension to the floor of the mouth (Figure 6).The excisional biopsy of both lobes revealed features concurrent with the incisional biopsy.

## Discussion

Peripheral Ossifying Fibromas are relatively common gingival growths, which are considered to be reactive in nature (2). In 1982, Gardner described the nature of the POF, which had earlier been considered the extraosseous counterpart of the central ossifying fibroma (4). Plaque, calculus, rough restorations, ill-fitting dentures, microorganisms, masticatory forces, minor trauma, trapped food and debris, and iatrogenic factors all influence the development of these lesions. In most instances, a POF lesion is associated with radiographic signs and tooth migration (5). Hence, POF is considered not to be neoplastic, andrather a hyperplastic reaction due to inflammation (6).

Although POFs are generally less than 2 cm in theirmaximum dimension (1, 2), larger atypical presentations have been documented. Such lesions have been referred to in the literature by several names, such as giant, large, atypical, huge, gigantiform POFs (7-16). Childers et al. in 2013 proposed the usage of a uniform standard terminology ‘Giant POF’ for atypical POFs to facilitate proper documentation of such presentations (3).

The clinical and radiographic features of GPOFs against that of conventional POFs have been tabulated in Table 1.

**Table 1:** Clinical and Radiological Features of Conventional Versus Giant Peripheral Ossifying Fibromas (1, 2, 3, 17

Features	Conventional Peripheral Ossifying Fibromas	Giant Peripheral Ossifying Fibromas
Age	Any age; more common in children and young adults	7.6 to 70 years
Site	Maxilla=Mandible; anterior to molar area	Mandible predominant (70%)
Clinical appearance	Well demarcated focal mass in gingival	Facial asymmetry; large, lobular growth; obliteration of vestibule
Base	Pedunculated/ sessile	Mostly pedunculated
Size	Maximum dimension – less than 2 cm	Maximum dimension - 2.5 to 9 cm
Duration to reach the dimension	Few weeks to months	1 month to 6 years
Radiographic features	No apparent underlying bone involvement	Displacement of vital structures seen in MRI; teeth displacement; no root resorption
Recurrence	Lesions recur. Repeated recurrences are not uncommon	1/10 case showed recurrence after 2 months; 6 cases showed no recurrence in follow up time ranging from 2 to 120 months.

Larger lesions with an acute onset (12) similar to the currentcasehave been very rarely reported.

The clinical, radiographic, and histological features of GPOFs reported in literature have been tabulated in Table 2.

**Table 2:** Clinical, Radiographic and Histological Features of Giant Peripheral Ossifying Fibromas Reported in the Literature

S. No.		Author(s)	Terminology Used	Age (in years)	Location	Size (in cm)	Base	Radiographic Features	Histological Features
1	**Thierbach et al. (2000)** ^[11^^]^		Atypical	23	Mandibular posterior	3	Pedunculated	Visible calcifications with no bone resorption	Fibrous stroma with ossifications
2	**Moon et al.** ** (2007) ** ^[10]^		Large	12	Maxillary anterior	3.5	Pedunculated	Visible calcifications with no bone resorption	Fibroblastic stroma with ossifications
3	**Kim and Kim** ** (2009) ** ^[15]^		Huge	66	Mandibular posterior	8	Pedunculated	Calcifications with bone resorption	Fibrous stroma with ossifications
4	**Poonacha et al. (2010) ** ^[12^^]^		Large	12	Maxillary posterior	2.5	Pedunculated	No calcifications/ Bone resorption	Fibroblastic stroma with ossifications
5	**Chaudhari and** **Umarji (2011) ** ^[14]^		Large	55	Mandibular posterior	5.9	Pedunculated	Calcifications with no bone resorption	Fibroblastic stroma with ossifications
6	**Trasad et al. ** **(2011) ** ^[16]^		Large	10	Maxillary posterior	6	Pedunculated	Calcifications with no bone resorption	Fibrous stroma with ossifications
7	**Sacks et al. (2012) ** ^[13]^		Gigantiform	52	Mandibular posterior	10.5	Pedunculated	No calcifications with focal bone resorption	Fibro- myxoidstroma with ossifications
8	**Childers et al. ** **(2013) ** ^[3]^		Giant	54	Mandibular anterior	4.5	Pedunculated	No focal resorption with calcification	Fibrous stroma with ossifications
9	**Present Case**		Giant	55	Mandibular posterior	5	Pedunculated	Soft tissue opacification with focal bone resorption	Fibroblastic stroma with ossifications

This atypical clinical presentation led to the impression of an aggressive or malignant lesion leading to the wide range of differential diagnoses, which were considered before the histopathological confirmation was obtained.

While it is well accepted that POF is most likely a reactive lesion, few cases of GPOF are known to clearly establish the pathologic process (3). 

Amongst reactive lesions of the oral cavity, POFs show higher mast cell count compared to pyogenic granuloma and peripheral giant cell granulomas (18). It has been reported that mast cells play a role in normal angiogenesis and in pathological angiogenesis that occurs in inflammatory diseases and tumors (19).

More studies of GPOF have to be conducted to clarify recurrence, pathologic process, and establish 

whether GPOF has sufficiently distinct characteristics from POFs. 

## Conclusion

Although GPOFs are rare, such lesions have to be considered during differential diagnoses even for huge lesions with acute onset similar to the present case. Incisional biopsies should be performed for such lesions in order to obtain a confirmatory diagnosis, which will aid abundantly for treatment planning. Biopsy technique for the sampling of such lesions can be critical. Care should be taken to harvest tissue fromthe deepest possible part of the lesion, as thisis necessary for diagnosis of such lesions.
